# Extrinsic Factors Driving Oligodendrocyte Lineage Cell Progression in CNS Development and Injury

**DOI:** 10.1007/s11064-020-02967-7

**Published:** 2020-01-29

**Authors:** Maryna Baydyuk, Vivianne E. Morrison, Phillip S. Gross, Jeffrey K. Huang

**Affiliations:** 1 Department of Biology and Center for Cell Reprogramming, Georgetown University, Washington, DC 20057 USA; 2 Interdisciplinary Program in Neuroscience, Georgetown University, Washington, DC 20057 USA

**Keywords:** Oligodendrocytes, Oligodendrocyte precursor cells (OPCs), Myelin, Demyelination, Remyelination, Diffuse white matter injury, Multiple sclerosis

## Abstract

Oligodendrocytes (OLs) generate myelin membranes for the rapid propagation of electrical signals along axons in the central nervous system (CNS) and provide metabolites to support axonal integrity and function. Differentiation of OLs from oligodendrocyte progenitor cells (OPCs) is orchestrated by a multitude of intrinsic and extrinsic factors in the CNS. Disruption of this process, or OL loss in the developing or adult brain, as observed in various neurological conditions including hypoxia/ischemia, stroke, and demyelination, results in axonal dystrophy, neuronal dysfunction, and severe neurological impairments. While much is known regarding the intrinsic regulatory signals required for OL lineage cell progression in development, studies from pathological conditions highlight the importance of the CNS environment and external signals in regulating OL genesis and maturation. Here, we review the recent findings in OL biology in the context of the CNS physiological and pathological conditions, focusing on extrinsic factors that facilitate OL development and regeneration.

## Introduction

The central nervous system (CNS) integrates and processes an immense amount of information leading to complex behavior. This multilevel process requires an extensive network of neural cell types to be established and maintained. During embryonic CNS development, progenitor pools of neuronal and glial cells expand and differentiate to acquire relevant functions. Oligodendrocytes (OLs) are glial cells whose terminal processes generate myelin and enwrap CNS axons. Myelin is crucial for saltatory propagation of electrical impulses down the axon, enabling rapid communication between networks of neurons in the CNS [1]. In addition to increasing axonal conduction speed through generating myelin, OLs secrete metabolic factors and maintain energy homeostasis to support axonal integrity and promote neuronal survival [2]. Thus, establishing proper numbers of OLs during brain development, as well as during their regeneration in neurological disorders that involve OL and myelin loss, is crucial for normal CNS function.

**Fig. 1:**
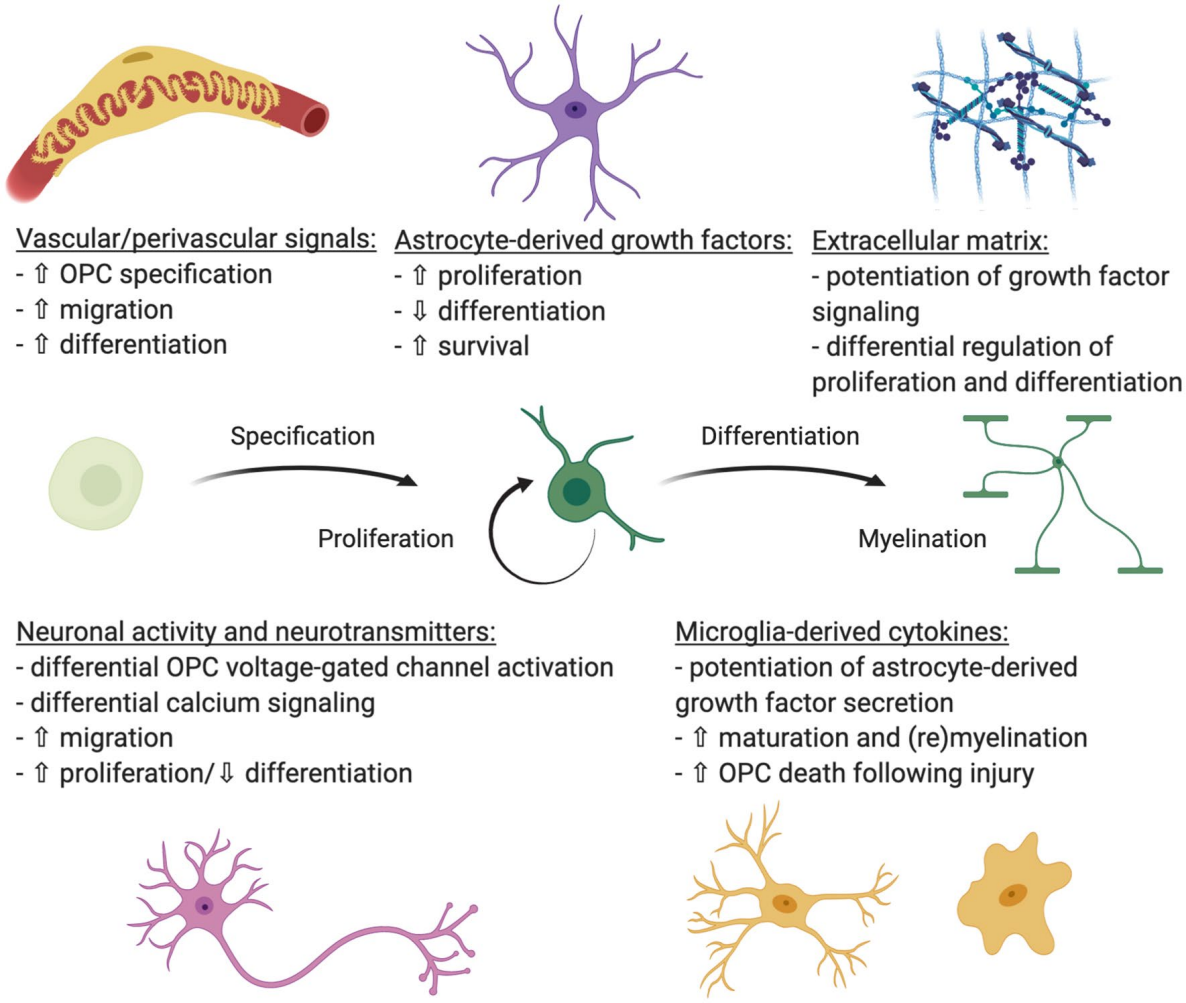
Extrinsic regulators of oligodendrocyte lineage cell progression from immature, migrating precursors to fully differentiated, myelinating oligodendrocytes

During CNS development, oligodendrocyte progenitor cells (OPCs) are generated from neural stem/progenitor cells (NSPCs) in several regions in a precise spatiotemporal manner [3–5]. Multiple transcriptional regulators cooperate to orchestrate changes in gene expression leading to OPC fate selection and subsequent differentiation to oligodendrocytes. One of the most important regulators of OL lineage cell development is oligodendrocyte transcription factor 2 (Olig2), which acts as a central node upon which numerous pathways converge and from which foundational intrinsic signals arise to drive OPC genesis and maturation [3, 4, 6–8]. For example, the Wnt/β-catenin and bone morphogenic protein (BMP) pathways inhibit *Olig2* gene function [9–11], while fibroblast growth factor (FGF), sonic hedgehog (SHH), retinoic acid (RA), and Notch1 signaling increase *Olig2* expression, facilitating OPC production, proliferation, and maturation [9, 12–14]. Once generated, transcriptional regulators such as Myrf, Myt, RXRs, are required for the differentiation of OPCs into oligodendrocytes [15–19]. Details of intrinsic signals regulating oligodendrocyte lineage cell specification and progression are well described elsewhere [4, 15, 16, 20] and will not be discussed further here. However, since OLs are part of an exquisitely complex CNS environment containing neurons, astrocytes, microglia, and vascular/perivascular cells, control of OL lineage cell proliferation and differentiation likely relies on multiple extrinsic cues and cell–cell interactions during development or regeneration. Here we will discuss the role of extrinsic factors in regulating the progression of OL lineage cells from immature, migrating precursors to fully differentiated, myelinating oligodendrocytes in development, aging, and disease (Fig. 1).

## Neuronal Regulation of OL Development

Since activity-dependent myelination was proposed over five decades ago [15], research focusing on neuronal regulation of OL development and function has revealed various pathways that might affect oligodendrocyte lineage progression from OPC proliferation to the terminal myelinating stage [15, 21–24]. Increasing evidence shows that neuronal activity and glutamate signaling can promote OPC migration, proliferation, differentiation, and myelination during development [21, 25–30]. OPCs receive synaptic inputs from neurons and express voltage-gated ion channels (such as voltage-gated sodium and potassium channels) and various neurotransmitter receptors [25, 27, 28, 31, 32]. Activation of voltage-gated sodium channels on OPCs leads to an increased number of proliferating OPCs and mature OLs [21, 33, 34], while potassium channel currents are known to enhance proliferation but may delay differentiation [32, 35, 36]. Glutamate released by excitatory neurons may serve as a chemoattractant, stimulating the migration of OPCs toward their target destination in the developing brain [26]. Activation of glutamate receptors on OPCs accelerates integrin-mediated OPC motility via mechanisms that involve AMPA receptors [26]. Glutamate signaling in OPCs also leads to potassium channel inhibition and decreased OPC proliferation [32]. The molecular mechanisms underlying glutamate signaling in OPCs involve elevated levels of the cell cycle inhibitors p27^Kip1^ and p21^Cip1^ [37–39], both of which naturally rise with continued OPC proliferation, constituting an “internal clock” or timing component of OL lineage cell progression and leading to stalling at the G1-S phase transition by dissociating cyclin-cdk complexes [35, 40–42]. These cell cycle inhibitors require thyroid hormone for their function and may, in turn, facilitate OPC maturation by triggering histone deacetylase-mediated changes in OPC gene function [42–46].

Activation of glutamate receptors on OPCs can also initiate local translation of myelin basic protein (MBP) and increase calcium transients in OPC/OL [47–49]. This process aligns with the observation that functionally active neurons are preferentially myelinated, while inhibition of glutamate release from a given axon decreases its likelihood of becoming myelinated [50–53]. It appears that some myelinated tracks rely on glutamatergic signals to induce myelination, while others do not. For instance, loss of vesicular glutamate release from reticulospinal neurons decreases their myelination, but similar loss in commissural ascending fibers has no impact on myelin [54]. This heterogeneity in the requirement of glutamate for myelination is not completely surprising, however, because while some myelinated regions contain exclusively glutamatergic projections (e.g. the corpus callosum), others contain a mixture of glutamatergic and GABAergic neurons. In addition to glutamate, OPCs can also respond to GABA. The impact of GABA signaling on OPCs depends on the type of GABA receptors that the cells express. For example, GABA_A_ receptor activation slows OPC proliferation and the extent of myelination, though curiously, the myelinated internodes formed by the GABA-stimulated cell are longer than those of non-GABA-stimulated cells [55]. Conversely, activation of GABA_B_ receptors stimulates OPC proliferation and migration [56]. The dynamics of axonal neurotransmitter release and receptor activation in OPCs are still largely unknown and further research is needed to fully uncover neuronal regulation of OL development.

Activity-dependent regulation of OL recruitment, lineage progression, and de novo myelination is evident from behavioral paradigms of motor learning. It has been shown that in mice learning to run on the complex wheel there is rapid differentiation of OPCs in the motor cortex, followed by a subsequent increase in compensatory proliferation to return to homeostatic OPC density [57–59]. Blocking OPC differentiation impedes motor learning and performance on the complex wheel [58]. Similarly, improvements in rodent spatial learning parallel increasing levels of OPC proliferation, myelination, and corpus callosum volume, and this effect is amplified by environmental enrichment [60]. Conversely, it has been demonstrated that social isolation of adult or early postnatal mice results in significant behavioral, transcriptional, and ultrastructural changes in OLs of the prefrontal cortex [61, 62]. Interestingly, these changes can be reversed by social reintegration in adult mice, however, the lack of social experience in juvenile mice during the critical period of prefrontal cortex development cannot be reversed by social reintroduction at later time points [61, 62]. These studies imply that activation of various neuronal circuits play an important role in white matter development (Fig. 1), while inhibition of neuronal activity may lead to myelin deficits [63].

## Role of Astrocytes in OL Maturation

Astrocytes precede OL in early postnatal development and regulate OPC proliferation, migration, survival, and differentiation through secreted factors such as PDGF, FGF, leukemia inhibitory factor (LIF), and a member of the interleukin (IL)-6 cytokine family [64–69]. When astrocytes fail to mature, i.e. in *Gfap* null mice, the lack of released growth factors cause white matter dysplasia and altered myelination [70], demonstrating that normal white matter development requires astrocytes. Mice with global PDGF deletion have a reduced number of OPCs and subsequent hypomyelination [71], whereas overexpression of PDGF in astrocytes of the mouse optic nerve leads to OPC hyperplasia [72]. Although, factors released by astrocytes often work in cooperation, they may also differentially regulate certain processes in OL development. For instance, PDGF applied in vitro prevents morphological maturation of OPCs, but cannot keep the cell in a constant proliferative state [73]. In contrast, FGF alone allows morphological maturation, but keeps the OPC in the cell cycle, albeit with a much-lengthened cell cycle time [73]. However, when both growth factors are present, OPCs can maintain an immature morphology and sustain proliferative capacity [73]. Similarly, the combination of PDGF and LIF promotes OPC survival, and while decrease in PDGF α-receptor signaling seems to be required for differentiation, continued LIF signaling in OLs stimulates myelination [68, 69].

Another soluble astrocyte-derived signal is endothelin-1 (ET-1), which has been shown to regulate OPC migration and differentiation [74]. Several isoforms of endothelins (ET-1, 2, and 3) are present in many tissues, including the brain at varying levels, and are also secreted by endothelial cells of microvasculature [75]. ETs induce their signaling through activation of their specific receptors (ETHRA and ETHRB), which are expressed by OPCs [74, 76]. Activation of ETHRs by ET-1 was found to play a dual role in early developmental stages in OL lineage progression by promoting OPC migration, while inhibiting their differentiation [74]. Interestingly, ET-1 alone did not promote OPC migration; instead it augmented the stimulatory effects of PDGF and FGF, possibly by facilitating the intracellular pathways activated by these growth factors in OPCs [74]. In contrast, ET-1 alone inhibited OPC progression to mature OLs by reducing differentiation of OPCs in the postnatal brain [74]. This observation is consistent with OL lineage progression, since OPC migratory potential decreases as they differentiate into mature myelin-producing OLs.

Astrocytes have also been shown to influence OL biology through secretion of extracellular matrix (ECM) proteins such as fibronectin and laminin [77]. Early in vitro studies demonstrated that fibronectin stimulated the migration and proliferation of OPCs via interaction with integrin receptors αvβ1 and αvβ3, respectively [78, 79]. Subsequent studies revealed an important role of a crosstalk between fibronectin and growth factor signaling by demonstrating that activation of the αvβ3 integrin receptor via fibronectin binding can potentiate the proliferative response elicited by the mitogenic growth factor PDGF-A [80, 81]. Moreover, both ECM proteins, fibronectin and laminin, have been shown to promote oligodendrocyte process extension by potentiating FGF2 [81, 82] in a protein kinase C (PKC)-dependent manner [83]. Taken together these and other studies demonstrate the importance of multiple astrocyte-derived factors on all aspects of OL biology (Fig. 1).

## Effect of Microglia on OPCs

Microglia are traditionally considered the resident immune cell population of the CNS, able to survey the tissue and attack pathogens and clear debris from normal, developmental apoptosis. We are just beginning to understand the integral role of microglia in brain development and function throughout life, specifically in the context of OL biology. A recent study shows that depletion of microglia ex vivo and in vivo does not affect OPC number in adult tissues, suggesting that microglia are not essential for OPC viability postnatally [84]. However, microglia activation, i.e. under injury conditions, appears to influence oligodendrocyte lineage cell progression [85]. Following activation with a proinflammatory stimulus, application of microglia-conditioned media (MCM) to myelinating co-cultures, while less effective at sustaining OPC viability than astrocyte-conditioned media, greatly increases the number of mature CC1^+^, MBP^+^ OLs and myelinated fibers [86, 87]. However, it is not possible to discern whether these pro-differentiation changes result from direct interactions of OPCs with microglial secreted factors or from indirect effects downstream of changes in other cell types such as astrocytes and neurons. The secreted factors in MCM that influence OPC development directly or through other cell types (such as astrocytes) appear to be largely cytokines and vary depending on the microglial activation state. For example, IL-1b, IL-6 family cytokines, and TNFa act through astrocytes to enhance OPC differentiation, by stimulating LIF transcription and secretion [88, 89]. Additionally, loss of TNFR signaling decreases CXCL12-CXCR4 interactions between astrocytes and OPCs, respectively, thereby decreasing OPC proliferation and differentiation [90].

In vivo, high numbers of activated microglia are observed in the SVZ between postnatal day 1 (P1) and P10, which corresponds to peak OPC specification from SVZ-resident NPSCs [91]. Similar observations were made in the postnatal corpus callosum (CC), where peak microglial numbers coincided with peak OPC turnover in the CC and many microglia were found to contain myelin debris [92], suggesting a role for microglia in subcortical white matter OPC homeostasis.

## Role of Vascular and Perivascular Cells in Oligodendrogenesis

Angiogenesis in the brain begins around embryonic day 10 (E10), when pericytes and other fibroblast-like cells and blood vessels penetrate the brain parenchyma, with the former developing into heterogeneous perivascular cell populations found in the postnatal brain [93–96]. Interestingly, angiogenesis in the CC continues well into the first two weeks of postnatal life, suggesting a connection between OPC development and the vasculature [97]. Indeed, there is a possibility that endothelial cells in the perineural vascular plexus surrounding the neurogenic niches of the forebrain [98] interact with neural precursors to drive OPC specification [99]. Similarly, endothelial cell-conditioned media provides trophic and pro-OPC specification cues for neural precursors and OPCs in vitro [100, 101]. The CNS vascular niche is composed of endothelial cells as well as perivascular cells that adhere to the blood vessels. It has been shown that pericyte-conditioned media applied to neurospheres increases *Olig2* mRNA and decreases transcription of the astrocyte-determinant *Id2*, ultimately favoring OPC specification over the astrocyte lineage [102]. Intriguingly, recent studies have found that OPCs use blood vessels as a scaffold for migration in the developing CNS [103]. The vascular and perivascular cells are located in close proximity to OPCs and can influence OPC proliferation and migration through both contact-dependent signals and secreted factors [103–105]. It was found that internalization of microvessel-derived extracellular vesicles promotes OPC survival, migration, and proliferation [106, 107]. This mechanism requires binding of vesicular protein fibronectin to heparan sulfate proteoglycan on OPCs [107]. Similarly, PDGF-dependent effects on OPC proliferation requires the expression of chondroitin sulfate proteoglycan 4/neural-glial antigen 2 (CSPG4/NG2) on the OPC cell surface [108–112]. Mice lacking NG2 fail to expand their OPC population [113, 114]. The epidermal growth factor (EGF) and vascular endothelial growth factor A (VEGFA) bind to their receptors on OPCs, EGFR, and VEGFR, respectively, and activate intracellular signaling, driving OPC migration [115–118]. Interestingly, there is a mutually beneficial relationship between OPCs and the vascular/perivascular population: OPCs regulate angiogenesis and pericyte colonization of the vasculature, while blood vessels and their associated cells support OPC proliferation, survival, and migration [97, 104, 117]. These studies also suggest that mild hypoxia in the perinatal white matter is not categorically detrimental, but rather may be a necessary part of OPC development and myelination, since loss of hypoxia-inducible factor signaling in OPCs leads to angiogenic failure and loss of white matter integrity in the developing brain [97].

Following demyelinating injury, OPCs are recruited into the lesion site via single cell perivascular migration on microvessels [119], similar to their developmental route, where OPCs require a vascular scaffold for their dispersal through the CNS. Recently, it has been shown that in multiple sclerosis (MS) lesions with active inflammation, OPCs can be found clustered on vasculature, representing a defect in single cell perivascular migration and inability to detach from blood vessels [119]. Interestingly, OPC perivascular clusters themselves can cause endothelial disruption and defects in blood–brain barrier integrity, triggering a subsequent CNS inflammation and contributing to pathology [119]. These findings suggest that oligodendroglial–vascular interaction is an important component regulating OPC migration and recruitment in developing and adult brain.

## Effect of Hypoxia on OPCs

Alterations in white matter development due to neonatal brain damage are often associated with significant delays and disruption of myelination, correlating with a period of maturation-dependent vulnerability of OPCs immediately before progressing to OLs [120–122]. One of the most common developmental impairments caused by hypoxia–ischemia in premature neonates is diffuse white matter injury (DWMI), which is associated with permanent neurological disabilities [123]. Identification of molecular mediators of OL regeneration in neonatal white matter following hypoxia is essential for developing therapeutic strategies to prevent neurodevelopmental deficits associated with this pathology in premature infants. Several studies using animal models of neonatal hypoxia–ischemia induced brain injury, which reproduce morphological and structural brain abnormalities found in DWMI, demonstrate biphasic changes in white matter, which start with OL death by apoptosis, followed by OPC proliferation during the first week after hypoxia, and resulting in delayed OL differentiation and abnormal myelination [123, 124]. The cellular and molecular mechanisms essential for the regenerative OPC responses after hypoxia involve activation of the Cdk2 signaling pathway, which promotes OPC proliferation [42, 125]; and reduction in expression level of p27^Kip1^ and its regulator FoxO1, resulting in delayed differentiation of OPCs [124]. In agreement with these findings in animal model, p27^Kip1^ was also reduced in OPCs found in human infant white matter lesions after hypoxia [124]. Later studies identified the histone deacetylase Sirt1 as a Cdk2 regulator of OPC proliferation in response to hypoxia [126]. Sirt1, which is specifically upregulated in proliferating OPCs after hypoxic insult, targets members of the Cdk2 pathway, causing epigenetic changes that drive Cdk2-mediated OPC proliferation [126].

Other studies have shown that signaling via epidermal growth factor receptor (EGFR) play important roles in OL development and regeneration [110, 118]. In mouse models of chronic neonatal hypoxia, a significant increase in the endogenous EGF levels was observed in the white matter [127]. Moreover, in these models, enhanced EGFR signaling stimulates the endogenous response of OPCs during a critical period after brain injury and promotes cellular and behavioral recovery in the developing brain [127]. Overexpression of human EGFR in oligodendrocyte lineage cells or the administration of intranasal EGF immediately after injury decreased oligodendroglia death, enhanced generation of new OLs, diminished ultrastructural myelin deficiency, and promoted functional recovery [127]. Since there are no clinically relevant treatments available to improve neurological outcomes of neonates with DWMI, molecular manipulations of the pathways that selectively enhance OL regeneration during a critical developmental time window after DWMI may serve as promising targets for promoting timely repair.

## Role of Inflammation in Oligodendrocyte Cell Lineage Progression

One of the most common demyelinating disorders of the adult CNS is multiple sclerosis (MS). In MS, damage to the white matter, caused by repeated immune-mediated attacks and destruction of myelin, results in neurodegeneration and progressive disability [128–132]. In the early stage of MS, endogenous CNS repair through remyelination takes place following demyelination and involves the recruitment, proliferation, and differentiation of oligodendrocyte precursor cells (OPCs) into myelin-producing oligodendrocytes [133, 134]. However, in the later, progressive stage, this regenerative process fails and most lesions remain demyelinated, leading to chronic axonal dysfunction and clinical deterioration [133–138]. The mechanisms that lead to remyelination failure in MS remain unknown. However, it has been shown that stalled OPC differentiation is caused by inhibitory signals present in the pathological lesion environment in patients with progressive MS [133, 135, 139]. Identification of these signals is essential to promote OPC differentiation and lesion repair.

The ability of progenitor cell populations to repair damaged tissue is modified by various growth factors, cytokines, and other intracellular signaling molecules, produced by many cell types present at the lesion site after demyelinating injury. During initial stages of demyelination, the highly inflammatory environment consists of reactive astrocytes, T-cells, pro-inflammatory macrophages and activated microglia, which secrete factors that promote OPC recruitment and proliferation [132]. At later stages of lesion progression, the inflammatory responses subside, resulting in OPC differentiation and myelin production [133, 134]. It is essential to understand how specific signals, produced by different cell types in the lesion, impact repair processes for further development of targeted approaches to enhance the beneficial responses that favor remyelination, while preventing the deleterious ones which inhibit it [139].

In addition to the previously discussed functions of astrocytes in OL development, astrocytes, depending on their activation state, can play an opposing role in OL remyelinating potential under injury conditions [77, 140]. Astrocytes display a continuum of phenotypes, ranging from the quiescent to more activated or reactive state, which can modulate myelination positively or negatively. For instance, in myelinating culture system, the presence of quiescent astrocytes, induced by tenascin C through CXCL10, results in less myelinated fibers [141], while activation of astrocytes through treatment with the cytokine, ciliary neurotrophic factor (CNTF), promotes myelination [141]. Conversely, astrocytes that have a severe reactive phenotype, induced by proinflammatory cytokines and CNS tissue damage, may secrete cytokines and chemokines that lead to myelin and oligodendrocyte damage, suppress remyelination, and delay disease recovery in animal models of demyelination [77, 140]. These studies demonstrate a direct correlation of astrocyte phenotypes with their ability to support remyelination and might have important implications with respect to the development of therapeutic strategies to promote CNS remyelination in demyelinating diseases.

Previous studies have shown that in addition to regulating OL development, astrocyte-derived factor, ET-1, also played an important role in OL repair responses after demyelinating insult, by inhibiting OPC differentiation and remyelination through activation of Notch signaling [142]. Previous studies have also shown that Notch1 inhibits OPC differentiation during both development and remyelination [143, 144]. Although astrocytes are not the only cells that produce and express ET-1, the largest increase in ET-1 expression was found in astrocytes following lysolecithin-induced focal demyelination and in MS lesions [142]. Moreover, infusion of exogenous ET-1 in mice during remyelination limited OPC differentiation, while selective genetic ablation of ET-1 in astrocytes significantly increased the number of mature OLs in focal demyelinated lesions and shifted the OL ratio from an immature to mature phenotype [142]. These findings indicate that astrocyte-derived ET-1 acts as an inhibitor of OPC differentiation and remyelination.

Endothelin receptors, EDNRA and EDNRB are upregulated after demyelination and are expressed by both reactive astrocytes and OPCs [74, 145]. However, it has been shown that ET-1 signaling through EDNRB, but not EDNRA, accelerates remyelination [146]. Moreover, selective EDNRB loss in astrocytes accelerated OPC differentiation, OL regeneration, and increased myelin production, whereas deletion of *Ednrb* in OPCs had no effect [146]. Together, these results demonstrate that reactive astrocytes indirectly inhibit OPC differentiation through ET-1 signaling.

The innate immune response to demyelination in the CNS that is comprised of peripherally derived macrophages and CNS residing microglia can potently influence OL differentiation and remyelination in the lesion [85]. The importance of macrophages/microglia is demonstrated by impaired remyelination following their depletion [147]. Two possible mechanisms, by which macrophages/microglia enhance remyelination are known: the clearance of myelin debris which is known to inhibit repair or secretion of regenerative factors such as cytokines [86, 148, 149]. Notably, macrophages/microglia can be polarized to distinct functional phenotypes: proinflammatory (M1) or anti-inflammatory/immunoregulatory (M2) [85, 86]. M1 ‘classically activated’ phenotypes are associated with enhanced antigen presentation properties and secretion of pro-inflammatory cytokines and reactive oxygen/nitrogen species, while M2 are thought to secrete anti-inflammatory cytokines/growth factors [150]. It has been proposed that as remyelination begins in the lesion, the switch from dominant M1 to M2 can occur within microglia and peripherally-derived macrophages. It has been shown that M2 macrophages/microglia are an essential part of an effective remyelination response, driving oligodendrocyte differentiation during lesion repair [86]. This M2-driven regenerative response is mediated, at least in part, by secretion of the TGFβ superfamily member, activin-A, and activates the mammalian target of rapamycin (mTOR) pathway, which has been previously implicated in positively regulating oligodendrocyte differentiation and/or myelination [85, 86, 151]. However, the M1 and M2 phenotypes represent a very simplistic view of macrophage/microglial phenotypes. More recent studies using newly developed technologies such as RNAseq, quantitative proteomics, and epigenetic approaches, have identified diverse populations of macrophages and microglia in health and disease, redefining our view of the complexity of immune cells in the CNS in physiological and pathological conditions [152–154]. These studies have characterized unique signature profiles of homeostatic and disease-associated subpopulations of tissue-derived macrophages and CNS microglia, uncovering their transcriptional identity and highlighting shifts in these population contributions in various neurological disorders.

Several macrophages/microglia populations have been shown to secrete the enzyme interleukin-four induced one (IL4I1), which is upregulated at the onset of inflammation resolution and remyelination [155]. Mice lacking *Il4i1* or its receptor show increased proinflammatory macrophage density, remyelination impairment, and axonal injury in the CNS lesions. Conversely, recombinant IL4I1 administration reduces proinflammatory macrophage density, enhances remyelination, and rescues remyelination impairment [155]. Remarkably, intravenous injection of IL4I1 into mice with experimental autoimmune encephalomyelitis (a widely used inflammatory mouse model of MS) at disease onset significantly reversed disease severity, resulting in motor function recovery [155]. These studies suggest that manipulating M2 polarization and secretion of pro-myelinating factors in the CNS lesion may present a complementary regenerative strategy to support remyelination and clinical recovery in MS.

Increasing evidence suggests that the immune cytokine interferon-gamma (IFN-γ), secreted by activated T-lymphocytes, plays a deleterious role in immune-mediated demyelinating disorders including MS. Although, normally excluded from the CNS, T cells enter the CNS through a compromised blood–brain barrier in these disorders and have been shown to inhibit OL differentiation and remyelination through cytokine receptors expressed by OPCs [156–159]. Recent study demonstrates that IFNγ induces expression of the MHC class I antigen presentation pathway in OPCs [160]. When exposed to IFNγ, OPCs switch from the constitutive proteasome to the immunoproteasome and are able to activate CD8+ T cells, which can in turn kill the OPCs as target cells, both in vitro and in vivo [160]. In addition, several other reports show that OPCs and oligodendrocyte lineage cells in MS express transcripts associated with inflammation and antigen presentation [161, 162]. These findings reveal that under inflammatory conditions OPCs responding to local cues may not only fail to differentiate, but could actually propagate chronic inflammation. Thus, strategies targeting the aberrant immune activation pathways in OPCs may allow more efficient remyelination in MS.

## Effect of Aging

Like the rest of the CNS, OLs and OPCs undergo phenotypic and functional changes with age. White matter volume begins to decline at ~ 45 years of age and aged OPCs in both humans and mice lose all or most of their ability to spontaneously remyelinate following demyelination [163–166]. As OPCs age, their excitability declines as a result of reduced NMDARs and voltage-gated sodium channel densities [167]. Markers of aging and senescence have also been reportedly localized to oligodendroglia in normal aging [163, 167] and in age-related neurodegenerative diseases [138, 164, 168]. These changes have been implicated to have a significant pathogenic role in diseases, including MS, Alzheimer’s Disease (AD), dementia, and amyotrophic lateral sclerosis (ALS) [2, 163, 164, 169]. In ALS, a recent study has shown an energetic dysfunction in oligodendrocyte-axonal coupling and failure of new oligodendrocytes to mature, resulting in demyelination [170]. In AD, Zhang et al. demonstrated that senescent cells associated with the amyloid-β plaques in AD patients were almost exclusively OPCs [168]. Interestingly, in a mouse model of AD treated with senolytic therapy that removed the senescent OPCs, they were able to ameliorate the amyloid-β associated inflammation and cognitive deficits.

Several studies implicated intrinsic regulators governing OPC aging, which is thought to be partially due to altered epigenetic modifications [165, 171–174]. While others strongly suggest the environment as the primary driver of oligodendroglial aging [63]. Like most cells, OL lineage cells are sensitive to different signaling factors that may change with age. Indeed, in vivo exposure of aged OPCs to youthful growth factors through parabiosis experiments has been found to significantly improve their ability to remyelinate after experimental demyelination [149]. Similarly, aged OPCs transplanted ex vivo to synthetic scaffolds mimicking the mechanical stiffness of young extracellular matrix’s (ECM) led to markedly increased proliferation and differentiation, while young OPCs transplanted to stiffer scaffolds mimicking aged ECM’s resulted in the loss of their capacity to proliferate and differentiate [175]. Additionally, dysfunctional protein aggregation and clearance within the aged environment has also been shown to induce senescent OPC phenotypes, which then directly contribute to disease pathogenesis [168]. Recent studies have shown that disruption of myelin debris uptake from young macrophages impairs the rate of remyelination similarly to those seen in older mice [176]. Indeed, aged phagocytes accumulate excessive amounts of myelin debris, owing to cholesterol crystal formation, which results in maladaptive immune responses downstream that limit OPC differentiation [166, 177]. Interestingly, by using a heterochronic parabiosis model, Ruckh et al. was able to facilitate enhanced remyelination in older mice to that comparable to their younger counterparts by recruitment of younger macrophages that enhanced the clearance of myelin debris following demyelination [149]. Since aged OL lineage cells are implicated in many neurodegenerative diseases, determining and reversing their aging mechanisms has tremendous therapeutic potential in the repair of demyelinating diseases like MS.

## Concluding Remarks

As we reviewed here, recent advances in OL biology have provided mechanistic insights into how OLs develop and regenerate in response to extrinsic signals. Heterogeneity in the OPC and OL populations adds another layer of complexity to the intricate, finely tuned regulation of myelination, which may have profound impacts on our brain function. The intrinsic differences in OPC populations may derive from their origins and include distinct transcriptomic profiles, ion channel expression and activity, varying across space and time [167, 171]. In the adult CNS, the survival of OLs is region specific and remyelination properties of OPCs are functionally diverse [171, 178]. Moreover, the contribution of putative subpopulations of oligodendrocyte lineage cells identified in the adult brain is shifted in MS [161, 162]. A recent study using snRNA-seq demonstrated a depletion of OPCs and the intermediate OL population, but increased expression of myelin genes in mature OL in MS, which may suggest that specific subsets of mature OLs contribute to remyelination [162]. This finding is further corroborated by recent work examining the retrospectively carbon (^14^C)-dated mature oligodendrocytes from post-mortem human brain tissue of healthy and MS patients [179]. The results of this study suggest that in MS lesions myelin may be regenerated by pre-existing, and not new oligodendrocytes. However, the contribution of mature oligodendrocytes to remyelination in animal models is still debated, most likely due to the degree of oligodendrocyte survival after demyelinating injury induced by different conditions [180, 181].

Our understanding of the complexity of OL biology in the developing and adult brain is likely to increase in the near future, as techniques for the analysis of differences in cell type and function evolve and improve. Precise and distinct regulation of important steps in remyelination, with consideration of the diversity of regenerative abilities among oligodendrocyte populations, will be essential for future strategies that aim to repair and restore brain function in neurological disorders.
